# Characteristics of syphilitic uveitis in northern China

**DOI:** 10.1186/s12886-017-0491-6

**Published:** 2017-06-19

**Authors:** Xiaomin Zhang, Qian Du, Feifei Ma, Yinglong Lu, Meiyan Wang, Xiaorong Li

**Affiliations:** 1 Eye Institute & School of Optometry and Ophthalmology, Tianjin Medical University Eye Hospital, 251 Fukang Road, Nankai District, Tianjin, 300384 China; 2 Department of Ophthalmology, Haibin People’s Hospital of Tianjin, Tianjin, 300280 China; 3 Department of Uveitis & Ocular Immunology, Eye Institute & School of Optometry and Ophthalmology, Tianjin Medical University Eye Hospital, 251 Fukang Road, Nankai District, Tianjin, 300384 China

**Keywords:** Syphilitic uveitis; chorioretinitis, Posterior placoid chorioretinitis, Pseudoretinitis pigmentosa, Neuroretinitis

## Abstract

**Background:**

To describe the characteristics of patients with syphilitic uveitis in northern China.

**Methods:**

A case series of 21 patients (33 eyes) diagnosed with syphilitic uveitis from 2011 to 2016 at a tertiary center in northern China was retrospectively analyzed.

**Results:**

Twenty-one patients (33 eyes) were diagnosed as syphilitic uveitis. Posterior segment involvement was found in 32 eyes (97.0%). Vitreous haze, neuroretinitis, and posterior placoid chorioretinitis were mainly found in patients with a relatively short duration of the disease, while diffuse chorioretinitis, pseudoretinitis pigmentosa, cystoid macular edema, and epiretinal membrane were found in patients with relatively long duration of ocular involvement. A low best-corrected visual acuity (*P* = 0.022) and a delay of syphilis treatment (*P* < 0.001) were associated with a significantly worse visual outcome.

**Conclusions:**

Syphilitic uveitis should be included in the differential diagnosis of any form of posterior ocular inflammation. The pattern of ocular involvement may change with the disease progression.

## Background

Syphilitic uveitis has recently regained attention due to the worldwide resurgence of syphilis [1–12]. Syphilis is a sexually transmitted, systemic infection caused by the spirochete *Treponema pallidum*. The clinical course of syphilis is typically divided into four stages, including primary, secondary, latent, and tertiary stages. Ocular involvement in syphilis can occur at any stage except in the primary stage, but occurs most often during the secondary or latent stages [1]. Syphilitic uveitis is the most common site of ocular syphilis. The reported incidence of syphilis among patients presenting at uveitis referral centers has significantly varied between studies, ranging from 0.4% in a Chinese study [5] in 2012 to 8% in a study from New York in 1997 [13].

Syphilitic uveitis is described as a “great masquerader” for its variable clinical manifestations that mimic other types of uveitis, and is often misdiagnosed, resulting in severe ocular complications and permanent visual loss [1, 2]. The purpose of this study was to describe the demographic and clinical features of a cohort of 21 patients with syphilitic uveitis over a 5-year period in a tertiary eye care center in northern China, and to assess the association between clinical and demographic data and visual outcome.

## Methods

This was a retrospective review of all cases of syphilitic uveitis documented between March 2011 and March 2016 in a tertiary uveitis service at Tianjin Medical University Eye Hospital. Approval for this study was obtained from Ethics Review Committee of Tianjin Medical University Eye Hospital (No. 2016KY(L)-08). Demographic and clinical data were collected from patient records, including age, gender, race, ocular and systemic history, presenting complaint, best-corrected visual acuity (BCVA), ophthalmic examination findings, concomitant systemic findings, laboratory test results, treatments, and outcomes on follow-up. Visual acuities were converted to the logarithm of the minimum angle of resolution (logMAR) for data analyses. The BCVA of counting fingers was recorded as 20/2000 for logMAR calculations.

A diagnosis of syphilitic uveitis was based on both serological tests and the efficacy of antibiotic therapy. Syphilis was confirmed using rapid plasma reagin (RPR) and Treponema pallidum particle agglutination (TPPA). Lumbar puncture was performed if neurosyphilis was suspected. All patients were also tested for human immunodeficiency virus (HIV) antibody. Ophthalmic examinations included Snellen visual acuity, slit lamp examination, tonometry, fundus biomicroscopy, and ancillary investigations, such as fundus fluorescein angiography (FA) and optical coherence tomography. Classification of uveitis was based on anatomical locations according to the Standardization of Uveitis Nomenclature Working Group criteria [14], and the criteria used by Villanueva et al. [15], with some modifications. The major criteria for the classification of posterior involvement used in our study are summarized in Table 1. All patients were referred to infectious disease specialists for further diagnosis and treatment.

**Table 1:** Classification of posterior syphilitic uveitis

Pattern	Description
Chorioretinitis	The inflammation of the choroid, retinal pigment epithelium and retina, with change of choroid perfusion, pigment epitheliopathy and retinal hyperfluorescence in fluorescein.
Neuroretinitis	The inflammation is confined to both retina and papilla optica.
Retinitis	The inflammation is confined to retina.
papillitis	The inflammation mainly affects papilla optica.
Primary retinal vasculitis	The inflammation mainly affects the retinal vessels.
Posterior placoid chorioretinitis	Fundus shows a yellowish, placoid, outer retinal lesion, involving the macular, with hyperfluorescence in the area of the lesion in fluorescein.

Influences of age, eye, gender, duration of the disease, previous systemic treatment, and initial BCVA on the prognoses were analyzed using SPSS statistical software for Windows, version 17.0 (SPSS, Chicago, IL, USA). Student’s *t*-test was used to analyze the differences of the means of final BCVA caused by gender, eye and previous systemic treatment, and Spearman’s rank correlation and Pearson linear correlation were used to correlate age, duration of the disease and initial BCVA with final BCVA. Finally, all variables were included in multiple linear regression models to identify independent risk factors that correlated with final BCVA. The eye was the unit of calculation and a *P* value ≤0.05 was considered to be statistically significant.

## Results

Twenty-seven patients were diagnosed with ocular syphilis, accounting for 1.9% of the total number of uveitis and scleritis patients that were referred to our department during the 5-year study period. Among these patients, 21 patients presented with syphilitic uveitis and six patients presented with scleritis. Only the documents of the 21 patients (33 eyes) with syphilitic uveitis were reviewed in this study. Both RPR and TPPA were positive in 20 patients. One patient had a negative RPR test, but a positive TPPA. Only one patient was HIV positive. The mean age at presentation was 44.9 years (range 20–64 years; median, 48 years) (Table 1). Eight patients (38.1%) were male and 13 patients were female (61.9%). Thirteen patients (61.9%) had a history of systemic manifestations of syphilis according to both the interview and the physical examination, most of which were mucocutaneous. Syphilis was first diagnosed in 18 patients (85.7%) during their first visit to our department, and only two male and one female patient were previously diagnosed with syphilis. Nine patients (42.9%) were diagnosed with secondary syphilis, two patients (9.5%) were diagnosed with neurosyphilis by lumbar puncture and 10 patients (47.6%) were diagnosed with a latent stage of syphilis. The duration between the initial symptom and the first presentation at our department ranged from 6 days to 24 months (mean 5.8 months; median, 6 months). The duration of 10 patients (47.6%) was longer than 6 months, while only that of two patients (9.5%) was less than 1 month. Twelve patients (57.1%) presented with bilateral uveitis.

**Fig. 1:**
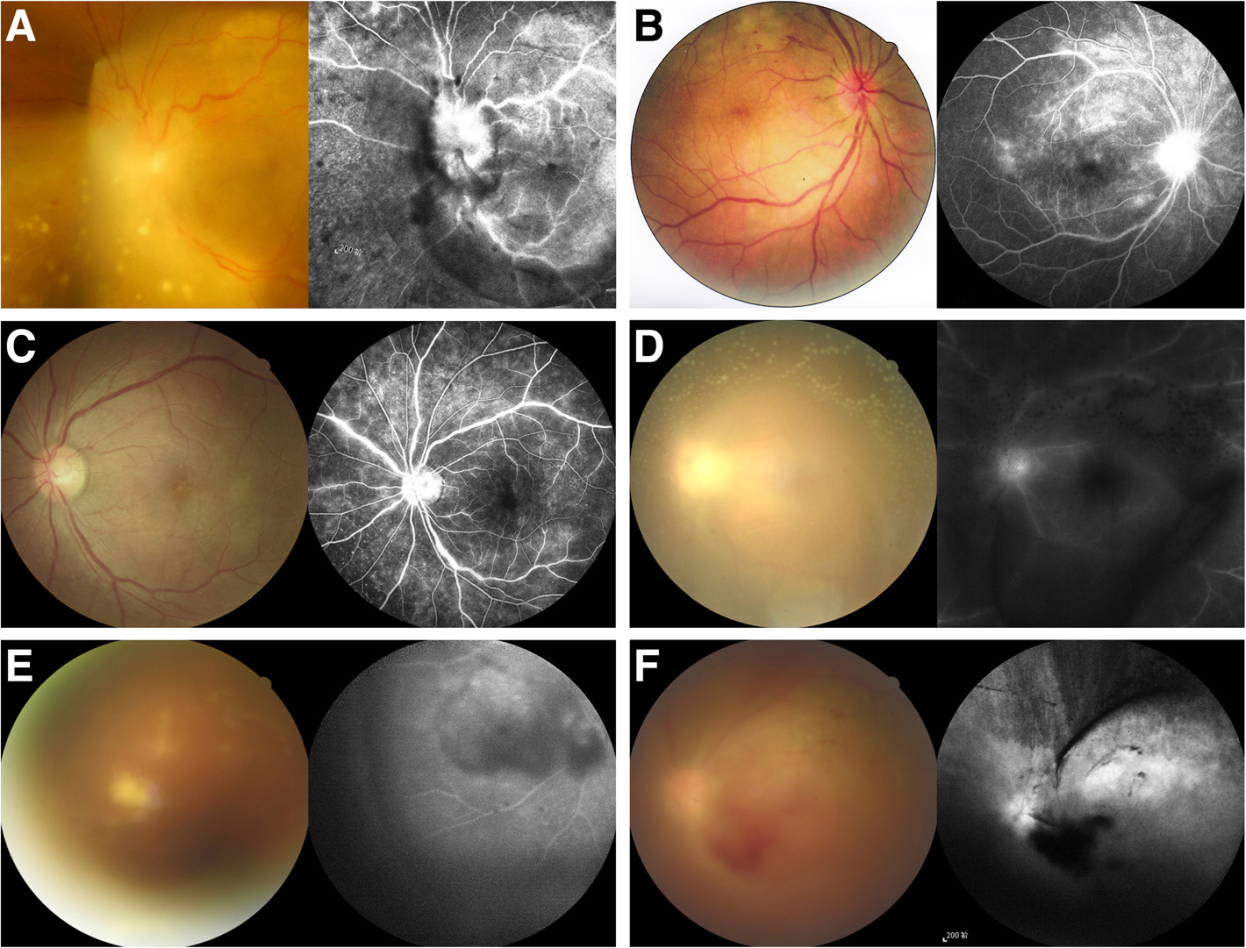
Typical fundus photographs and fluorescein angiographies. **a** diffuse chorioretinitis with multiple white-yellow punctuate nodules on the retina; **b** neuroretinitis; **c** vasculitis; **d** severe vitreous haze and multiple mutton fat-like inner precipitates; **e** severe vitreous haze and white-yellowish masses attached to the peripheral posterior vitreous membrane; **f** severe vitreous haze and vitreous hemorrhage

**Fig. 2:**
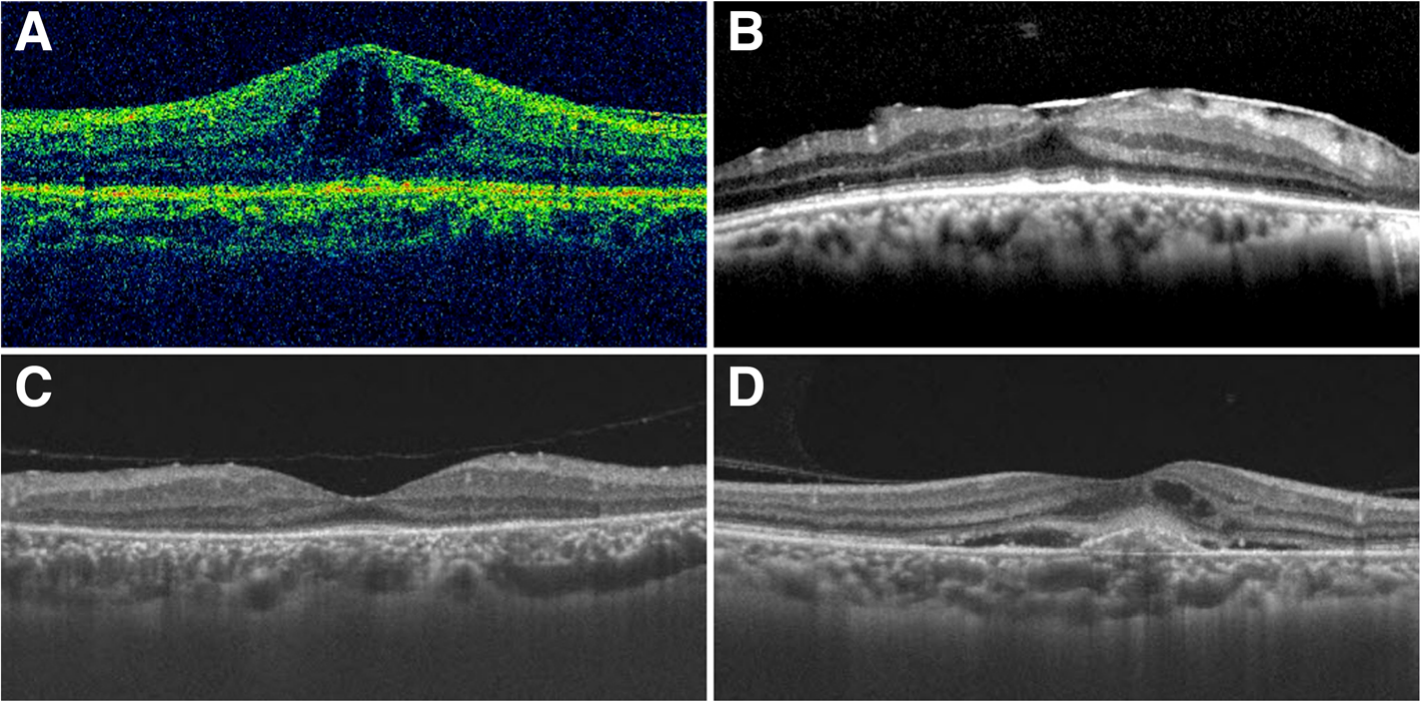
Typical pictures of macular changes. **a** cystoid macular edema; **b** epiretinal membrane; **c** disappear of the photoreceptor inner segment-outer segment junction line; **d** choroidal neovascularization

Clinical features of the 33 eyes are summarized in Table 2. Blurring of vision was the most common complaint in 31 eyes (93.9%), followed by floaters (14 eyes, 42.4%), redness (11 eyes, 33.3%), pain (7 eyes, 21.2%), and photopsia (7 eyes, 21.2%). The initial BCVA ranged from no light perception (NLP) to 20/20, with a median of 20/70. It was 20/40 or better in nine eyes (27.3%), 20/50–20/200 in 14 eyes (42.4%), and worse than 20/200 in 10 eyes (30.3%). Of the 33 eyes studied, posterior uveitis was seen in 19 eyes (57.6%), panuveitis in 13 eyes (39.4%), and intermediate uveitis in one eye (3.0%). Only one eye (3.0%) presented with a granulomatous form. Episcleritis was found in one eye (3.0%), hypopyon was found in two eyes (6.1%), and posterior synechiae was found in one eye (3.0%). Mild to marked vitreous haze was noted in 13 eyes (39.4%). Diffuse chorioretinitis was seen in 19 eyes (57.6%), and was the most common type of posterior inflammation. Other types included neuroretinitis in seven eyes (21.2%), papilitis in two eyes (6.1%), vasculitis in one eye (3.0%), and posterior placoid chorioretinitis in three eyes (9.1%). The retinal complications included pseudoretinitis pigmentosa in 11 eyes (33.3%), retinal or vitreous hemorrhage in three eyes (9.1%), vascular occlusion in two eyes (6.1%), and inner precipitates on the posterior vitreous membrane in two eyes (6.1%). Multiple white-yellow punctuate nodules on the retina and patchy white-yellow lesions were noted in one eye (3.0%). Typical fundus photographs and FAs are shown in Fig. 1. Macular changes involved cystoid macular edema (CME) in six eyes (18.2%) and epiretinal membrane in four eyes (12.1%) (Table 2). A disruption of the photoreceptor inner segment-outer segment junction line was present in most cases. Choroidal neovascularization (CNV) developed after a relapse of inflammation in one eye (patient 2). Figure 2 shows the typical pictures of macular changes.

**Table 2:** Summary of demographic and clinical history of syphilitic patients (*n* = 21)

Demographic and clinical history	No. (%) of patients
Gender	
Male	8 (38.1%)
Female	13 (61.9%)
Clinical findings	
Systemic and ocular syphilis	13 (61.9%)
Ocular syphilis only	9 (42.9%)
Evidence of neurosyphilis	2 (9.5%)
Diagnosis of syphilis	
Known case	3 (14.3%)
Male	2 (9.5%)
Female	1 (4.8%)
Diagnosed after presentation	18 (85.7%)
Male	6 (28.6%)
Female	12 (57.1%)
Stages	
Primary syphilis	0
Secondary syphilis	9 (42.9%)
Latent syphilis	10 (47.6%)
Tertiary syphilis	2 (9.5%)
Duration	
≥ 6 months	10 (47.6%)
	9 (42.9%)
	2 (9.5%)
≤ 1 month; < 6 months	
< 1 month	9 (42.9%)
	12 (57.1%)
Eyes involved	
Unilateral	13 (61.9%)
Bilateral	8 (38.1%)
Previous diagnosis	
Misdiagnosed	7 (33.3%)
Undiagnosed	3 (14.3%)
Usage of systemic immunosuppressants	1 (4.8%)
≥ 2 months	10 (47.6%)
≤ 0.5 month; < 2 months	
< 0.5 month	
Without systemic therapy

**Fig. 3:**
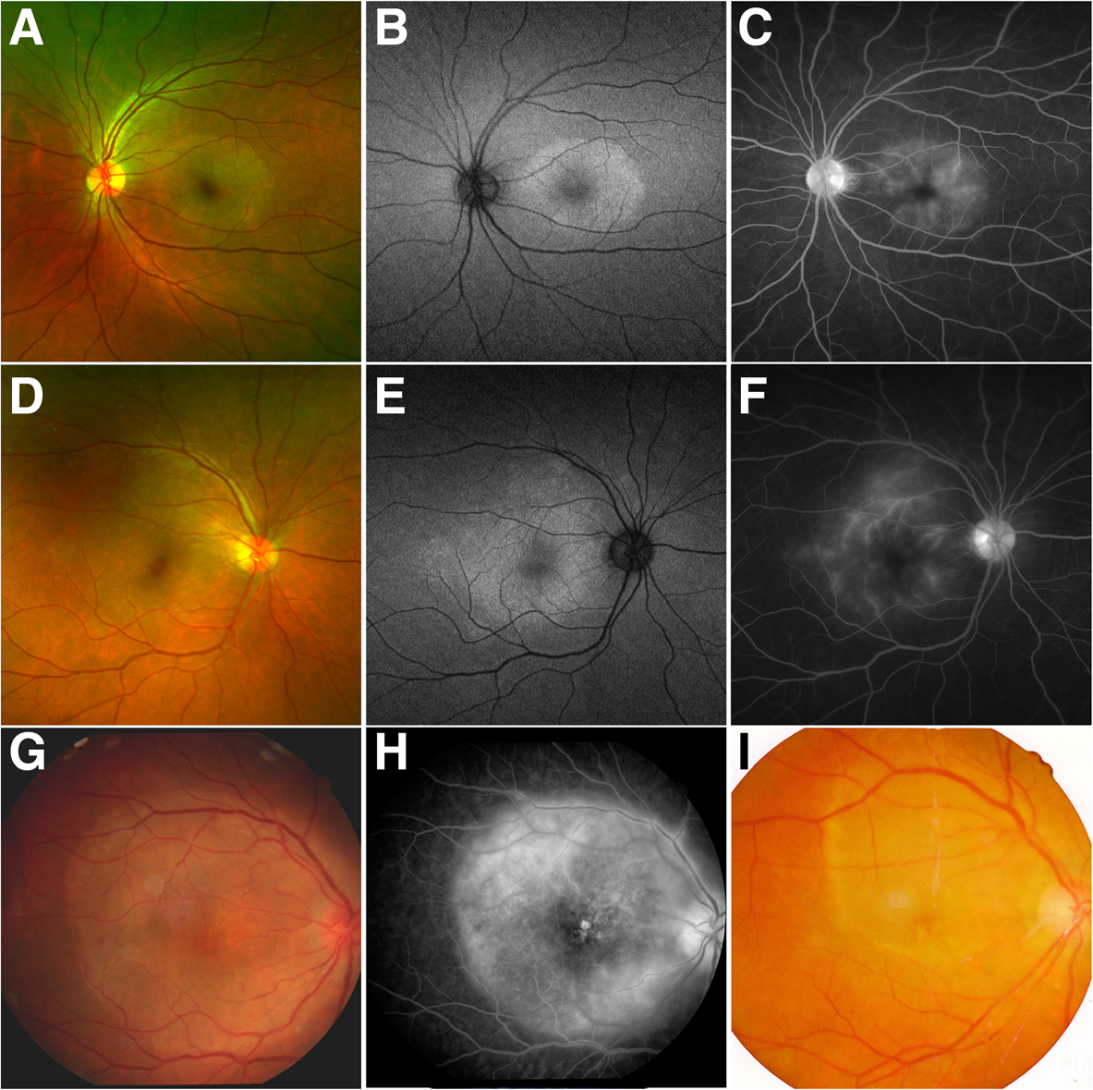
Fundus manifestations of posterior placoid chorioretinitis. **a** fundus photograph of the left eye of patient 5; **b** autofluorescence image of the left eye of patient 5; **c** fluorescein angiography of the left eye of patient 5; **d** fundus photograph of the right eye of patient 5; **e** autofluorescence image of the right eye of patient 5; **f** fluorescein angiography of the right eye of patient 5; **g** fundus photograph of the right eye of patient 19; **h** fluorescein angiography of the right eye of patient 19; **i**: fundus photograph of the right eye of patient 17 taken two months ago before visiting our department

Posterior placoid chorioretinitis was found in three eyes of two patients. The eyes were observed to have a yellowish, placoid, outer retinal lesion, involving the macula (Fig. 3a–i). FA showed hyperfluorescence in the area of the lesion (Fig. 3c–h). Autofluorescence imaging was performed in the patient with bilateral posterior placoid chorioretinitis, which revealed an increased autofluorescence in the lesion area (patient 5; Fig. 3b, e). The other patient had signs of episcleritis, without evidence of anterior or posterior scleritis (patient 19; Fig. 3g, h). Of note is that the fundus image of patient 17 at the onset of the disease showed the same change of the retina as posterior placoid chorioretinitis (Fig. 3i). After 2 months of corticosteroid therapy before visiting our department, the visual acuity of the abnormal eye temporarily improved, but subsequently worsened. Fundus examination revealed moderate vitreous haze, diffuse retinal, and choroidal inflammation (Fig. 1e).

**Fig. 4:**
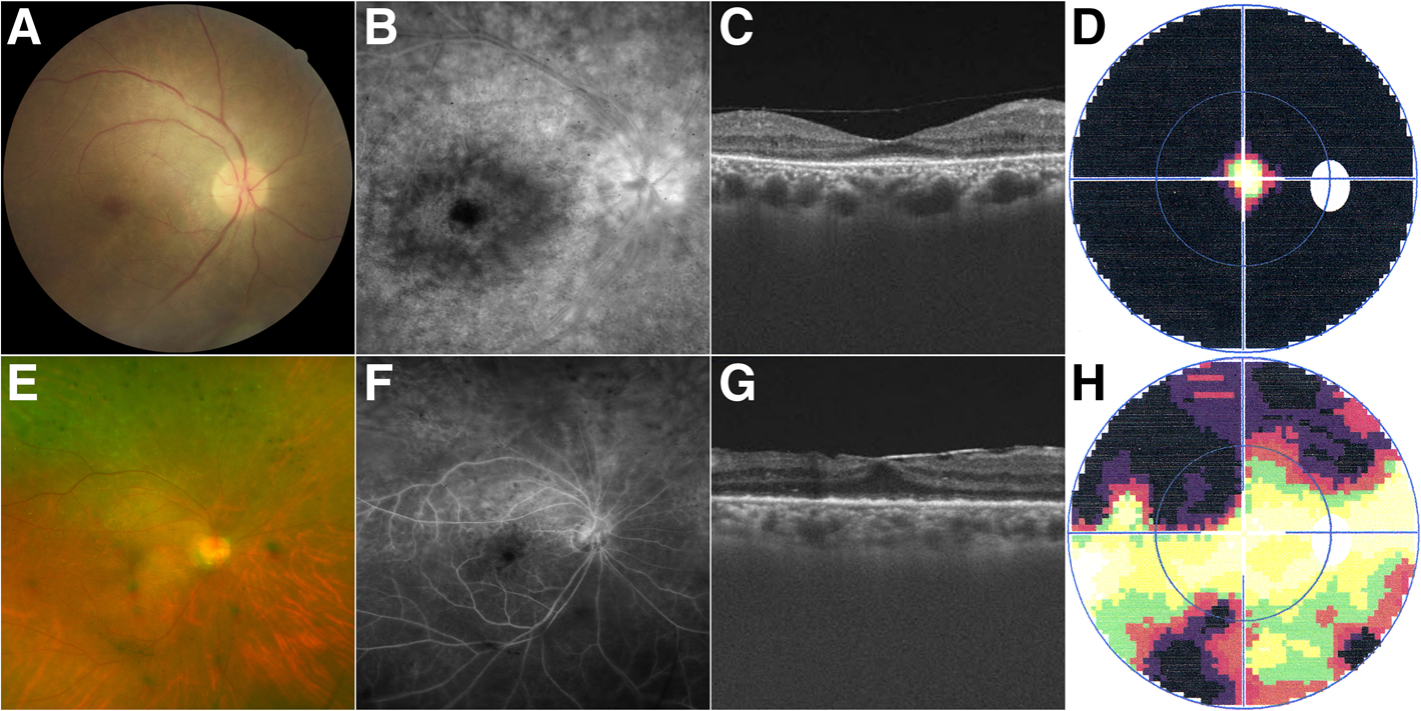
Typical pictures of pseudoretinitis pigmentosa. The fundus photograph, fluorescein angiography, optical coherence tomography, and visual field of patient 4 (**a**-**d**) and 6 (**e**-**h**)

As shown in Tables 3 and 4, vitreous haze, neuroretinitis, and posterior placoid chorioretinitis were mainly present in patients with a relatively short duration of the disease, while chorioretinitis, pseudoretinitis pigmentosa, CME, and epiretinal membrane were found in patients with a long duration of the ocular involvement. Pseudoretinitis pigmentosa, a pigmentary retinopathy similar in appearance to retinitis pigmentosa (RP), was observed in patients with a duration over 6 months, with severe damage of the visual field (Fig. 4).

**Table 3:** Summary of clinical characteristics of eyes with syphilitic uveitis (*n* = 33)

Clinical characteristics	No. (%) of eyes
Presenting complaint	
Blurring of vision	31 (93.9%)
Floaters	14 (42.4%)
Redness	11 (33.3%)
Pain	7 (21.2%)
Photopsia	7 (21.2%)
Initial BCVA	
≥ 20/40	9 (27.3%)
20/50–20/200	14 (42.4%)
< 20/200	10 (30.3%)
Final BCVA	
≥ 20/40	21 (63.6%)
20/50–20/200	11 (33.3%)
< 20/200	1 (3.0%)
Improved	27 (81.8%)
Unchanged	7 (21.2%)
worsen	1 (3.0%)
Type of uveitis	
Anterior uveitis	0
Intermediate uveitis	1 (3.0%)
Posterior uveitis	19 (57.6%)
Panuvetiis	13 (39.4%)
Granulomatous	1 (3.0%)
Nongranulomatous	12 (36.4%)
Ocular signs	
Episcleritis	1 (3.0%)
Hypopyon	2 (6.1%)
Posterior synechiae	1 (3.0%)
Vitreous haze	13 (39.4%)
Chorioretinitis	19 (57.6%)
Neuroretinitis	7 (21.2%)
Papilitis	2 (6.1%)
Vasculitis	3 (9.1%)
Posterior placoid chorioretinits	3 (9.1%)
Pseudoretinitis pigmentosa	11 (33.3%)
Retinal or vitreous hemorrhage	3 (9.1%)
Vascular occlusion	2 (6.1%)
Inner precipitates	2 (6.1%)
White-yellow punctuate nodules	1 (3.0%)
Patchy white-yellow lesions	1 (3.0%)
Cystoid macular edema	6 (18.2%)
Epiretinal membrane	4 (12.1%)

Note. *BCVA* best-corrected visual acuity

**Table 4:** Ocular signs and duration of disease (*n* = 33)

Ocular signs	Duration (month)		
	Range/mean	No. (%) of eyes	
		< 6 months	≥ 6 months
Vitreous haze	0.2–12/3.2	9 (27.3%)	4 (12.1%)
Choroidretinitis	1–24/7.3	5 (15.2%)	14 (42.4%)
Neuroretinitis	0.2–2/1.3	7 (21.2%)	0
Posterior placoid chorioretinits	1–2/1.3	3 (9.1%)	0
Pseudoretinitis pigmentosa	6–24/9.6	0	11 (33.3%)
Cystoid macular edema	2–12/10.0	1 (3.0%)	5 (15.2%)
Epiretinal membrane	6–12/9	0	4 (12.1%)

Thirteen patients (61.9%) were misdiagnosed and eight patients (38.1%) were undiagnosed before referral to our department (Table 1). Ten patients had been diagnosed with autoimmune uveitis without classification, one patient as Behçet’s disease, one patient as optic neuritis, and one patient as anterior ischemic optic neuropathy. Eleven patients (52.4%) had been treated with systemic immunosuppressants (Table 1) that were usually oral prednisolone, and seven patients (33.3%) had been treated for 2 months or longer (Table 1). The patient misdiagnosed with Behçet’s disease (patient 20) had been administrated 10 months of combined corticosteroids and immunosuppressants, including methylprednisolone, prednisolone, cyclophosphamide, and chlorambucil. She visited our clinic after the fourth attack of ocular inflammation, with a visual acuity of NLP in her right eye. Fundus examination revealed severe chorioretinitis and vasculitis with vascular occlusions and white-yellow nodules scattered on the peripheral retina (Fig. 1e). Her RPR test was negative, while TPPA was positive. The ocular inflammation subsided rapidly after antibiotic therapy, leaving permanent vision loss of her right eye.

Most patients were treated with either daily intravenous penicillin G for 14–15 days or weekly intramuscular injections of benzathine penicillin G for 3 weeks (patient 2). One patient was allergic to benzathine penicillin G and received oral tetracycline for 2 weeks. Relapse occurred in this patient 3 months after the first therapy, with slight vitreous inflammation and CNV formation. Patients with anterior inflammation were given topical steroids. Oral prednisone was prescribed to all patients except patients 20 and 21, at a low and tapering dose, with a range of 15–40 mg. Ocular inflammation resolved rapidly after treatment with antibiotics in all patients. CME persisted in three eyes of two patients after antibiotic therapy, and partly resolved after sub-Tenon’s capsule injection of triamcinolone. The CNV that developed after a relapse responded to combined intravitreal injection of triamcinolone and anti-vascular endothelial growth factor. The mean follow-up time was 5.1 months (range, 1–24 months). The final BCVA was 20/40 or better in 21 eyes (63.6%), 20/50–20/200 in 11 eyes (33.3%), and worse than 20/200 in one eye (3.0%), and the median was 20/40. The BCVA improved in 27 eyes (81.8%), was unchanged in seven eyes (21.2%), and worsened in one eye (3.0%) because of CNV formation.

Univariate analyses showed that the systemic immunosuppressive therapy prior to the start of antibiotic treatment (*P* = 0.019) was associated with worse final BCVA (Table 5). The duration of the disease (*r* = −0.518, *P* = 0.002) and the age (*r* = −0.512, *P* = 0.002) negatively correlated with the final BCVA, while the initial BCVA (*r* = 0.640, *P*<0.001) positively correlated with the final BCVA (Table 6). However, using multivariate linear regression, we found that only a low initial BCVA (*P* = 0.022) and a delay of syphilis treatment (*P* < 0.001) were associated with a significantly worse BCVA outcomes (Table 7).

**Table 5:** Associations of factors (dichotomous variable) with the final BCVA in univariate analyses

Variables		n	x ± s	t	p
Gender	Male	13	0.685 ± 0.339	1.059	0.298
	Female	20	0.561 ± 0.320		
Eye	OD	19	0.564 ± 0.313	−0.925	0.362
	OS	14	0.671 ± 0.350		
Systemic immunosuppressive therapy	Yes	14	0.457 ± 0.293	−2.467	0.019
	No	19	0.722 ± 0.313		

Eye based analysis (*n* = 33)

**Table 6:** Associations of factors (continuous variables) with the final BCVA in univariate analyses

Variables	r	p
Duration	−0.518^a^	0.002
Age	−0.512^b^	0.002
Initial BCVA	0.640^b^	<0.001

Note. *BCVA* best-corrected visual acuity, ^a^Spearman’s rank correlation was used, ^b^Pearson linear correlation was used

Eye based analysis (*n* = 33)

**Table 7:** Factors significantly associated with the final BCVA in multiple linear regression models

Variables	β	Stβ	t	p
Constant	0.505	—	6.246	<0.001
Duration	0.621	0.576	4.399	<0.001
Initial BCVA	−0.020	−0.316	−2.413	0.022

Note. *BCVA* best-corrected visual acuity

Eye based analysis (*n* = 33)

## Discussion

In this study, we summarized the features of syphilitic uveitis in a local population of northern China. Coinfection of syphilis and HIV was only observed in one patient, and the sexual history was difficult to assess in our study because of traditional Chinese customs. Compared with other reports [4, 10], the frequency of co-infection of HIV in our patients with syphilitic uveitis was much lower, which may be due to the low prevalence of HIV in China, especially in northern China [16]. There were more female patients than male patients in our cohort. This may have resulted because syphilis was more easily diagnosed in males in ophthalmological clinics than in women in a conservative city in northern China, and/or more female patients were regarded as refractory cases and were referred to our tertiary uveitis service. Systemic manifestations were found or retrieved in more than half of the patients, most of which were mucocutaneous. There is a general consensus that lumbar puncture should be performed on all the patients with syphilitic uveitis. However, most of the patients refused this examination due to both the cost and the risks of the operation. Considering the good prognosis of the disease and to avoid medical dispute, lumbar puncture was only performed on the patients with suspected neurosyphilis, and the results of two patients turned out to be positive.

In our department, all patients with unexplained uveitis were investigated for syphilis. Because the nonspecific test, RPR, is rapid and convenient, most of the patients were initially screened using RPR, and then confirmed by the specific test, TPPA. However, RPR was negative in patient 20, who was diagnosed as having Behçet’s disease before and at a latent stage of syphilis. Because the condition in her left eye continuously deteriorated after immunosuppressive therapy, TPPA was further examined and resulted in being positive. RPR is thought to be highly sensitive in the secondary stage, but not in the primary or tertiary stages [17]. It has been reported that use of RPR as a screening test misses 11% of patients with ocular syphilis who had a negative RPR test [18]. It is therefore inadequate to order a RPR test for a patient with suspected ocular syphilis.

All patients in our study had posterior involvement, and anterior segment inflammation with panuveitis was found in 39.4% of the eyes. Amaratunge et al. in 2010 reported in a literature review of 141 patients that posterior uveitis was the most common form of syphilitic uveitis in the Caucasian and African-American populations [19]. The other two reports from China reported that posterior involvement was also the main feature of ocular syphilis in the Chinese population [5, 12]. However, this was not the case in a study from Singaporean consisting of nine (75.0% of all patients) Chinese patients who were diagnosed with isolated anterior uveitis in 33.3% of the eyes [7]. These differences might be due to the different duration of the disease from onset to final diagnosis. The duration of the patients referred to our department ranged from 3 days to 24 months, with a mean of 5.8 months. Delayed treatment might lead to spreading of the ocular infection, for example, from the anterior to the posterior segment and from local to diffuse inflammation, thereby changing the uveitis pattern. Notably, patient 17 in our study had signs of posterior placoid chorioretinitis at the onset of the disease, but it was diagnosed 2 months later as diffuse chorioretinitis with severe vitreous haze.

The distribution of the type of posterior segment presentation has shown great variability in different studies. The difference may be due to both the discrepancy in duration of the disease as discussed above and the diversity of classification criteria. In our study, consistent with the report of Villanueva et al. [15], diffuse chorioretinitis (19 eyes, 57.6%) was the most common presentation, and was the only manifestation in patients with a duration >6 months, while in patients with a duration <6 months, neuroretinitis was the most common diagnosis. In addition, vitreous haze and posterior placoid chorioretinitis were other symptoms mainly observed in patients with short duration of the disease, while pseudoretinitis pigmentosa, CME, and epiretinal membrane were found in patients with long history of ocular involvement. These results also suggested that the infection might spread in the eye without effective therapy, and a typical severe disease might develop from local inflammation to diffuse chorioretinitis with vitreous haze, and finally result in pseudoretinitis pigmentosa. Antibiotic treatment can still improve the visual acuity at the later stages of the disease, but fails to broaden the visual field effectively once the outer layer of the retina and RPE is widely destroyed, emphasizing the importance of early diagnosis.

Posterior placoid chorioretinitis is an uncommon but distinct manifestation of ocular syphilis, which is characterized by a large, placoid, yellowish, outer retinal lesion with hyperfluorescence in FA and slightly increased autofluorescence [20, 21]. Eandi et al. reported a series of 16 cases with active posterior placoid chorioretinitis collected from 12 retina practices in the United States and Europe in 2012, and retrieved 44 previously reported patients, suggesting that all patients with signs of posterior placoid chorioretinitis should be tested for syphilis [22]. Two patients were diagnosed with posterior placoid chorioretinitis in our case series, who experienced good vision recovery after antibiotic therapy.

Consistent with recent reports, nongranulomatous inflammation was more frequent in our study. Granulomatous uveitis was observed in only one patient, with mutton fat KPs. The inner precipitates found in patient 17 were white-yellowish masses with varying sizes that were attached to the peripheral posterior vitreous membrane, while in patient 21 with a coinfection of syphilis and HIV, multiple mutton fat-like precipitates were observed. These kinds of precipitates have been previously described in several reports, and were thought to represent granulomas comprised of bacteria and inflammatory cells [5, 23–26]. Yang et al. suggested that these structures might be a diagnostic and/or predictive parameter for ocular syphilis [5].

Corticosteroid therapy can temporarily improve the symptoms of patients with syphilitic uveitis, and this improvement might be mistakenly regarded as an indication of proper treatment. However, syphilitic uveitis can sometimes subsequently worsen with the development of the disease [11, 17, 27]. It has therefore been suggested that corticosteroid should never be used alone to treat syphilis [3]. In our study, multivariate linear regression analyses revealed that only the duration of the disease and the initial BCVA independently correlated with a worse prognosis, while the usage of systemic corticosteroid treatment did not affect visual acuity outcomes independently. It has been reported in a recent report enrolling 85 patients that the mean visual acuity at the start of syphilis treatment was better in patients receiving steroid therapy, indicating a potential beneficial effect of steroid treatment [11]. The CME responded to periocular injection of steroids in our patients, indicating that steroids may be useful for controlling the inflammation when used together with effective antibiotic therapy. In addition, steroids have been suggested to be used together with antibiotic therapy to reduce the incidence of the Jarisch–Herxheimer reaction [28]. Taken together, we suggest a combination of steroid and antibiotic therapy for syphilitic uveitis, especially in severe cases.

## Conclusion

In conclusion, syphilitic uveitis is commonly overlooked in China, and should be included in the differential diagnosis of any form of posterior ocular inflammation due to its variety of clinical manifestation. The pattern of ocular involvement may change with the development of the disease. Neuroretinitis and diffuse chorioretinitis with vitreous haze are the typical presentations, which may result in pseudoretinitis pigmentosa without efficient therapy. Early treatment of syphilis will lead to a good visual prognosis, while a delay in therapy may lead to severe ocular complications.
